# Prevalent zoonoses in Sao Paulo State, Brazil: the role of bats and molecular diagnosis

**DOI:** 10.1590/S1678-9946202567017

**Published:** 2025-03-03

**Authors:** Danilo Alves de França, Helio Langoni

**Affiliations:** 1 Universidade Estadual Paulista, Departamento de Produção Animal e Medicina Veterinária Preventiva, Botucatu, São Paulo, Brazil

**Keywords:** Surveillance, Reservoir, Infectious agents, Zoonoses, Literature review

## Abstract

This review explores the landscape of prevalent zoonotic diseases in Sao Paulo State, Brazil, focusing on the role of bats as reservoirs and the application of molecular biology in the diagnosis. The zoonoses covered include visceral and cutaneous leishmaniasis, Chagas disease, toxoplasmosis, bartonellosis, Q fever, Brazilian spotted fever, and leptospirosis. Molecular techniques can improve public health responses by accurately identifying pathogens and tracking their transmission dynamics in populations, thus enhancing early detection, characterization of strains, and monitoring of disease outbreaks. By elucidating the epidemiology and molecular aspects of zoonoses associated with bats in Sao Paulo State, we highlight the importance of integrated surveillance systems and multidisciplinary approaches to effectively manage and prevent these diseases.

## INTRODUCTION

Brazil faces significant challenges related to zoonoses, which are infectious diseases transmitted between animals and humans. Bats are important reservoirs of zoonotic agents, being vital to better understand and mitigate these diseases. With advances in molecular biology, new diagnostic techniques have revolutionized the identification and study of these zoonoses, offering new perspectives to comprehend epidemiology and implement control measures^1^.

Many zoonoses are classified as public health issues in Sao Paulo State. Regarding prevalence, severity and lethality, leishmaniasis and leptospirosis are among the most significant zoonoses. Chagas disease and toxoplasmosis are also prevalent and, in specific cases, can also lead to death. Some other zoonoses also stand out in terms of severity and lethality, but are highly underdiagnosed, and recent studies have shown that they circulate in animals and humans in the statewide, such as Brazilian spotted fever, bartonellosis and Q fever^2^.

These diseases share common reservoirs, which participate in transmission cycles and their occurrence in humans. This is the case with dogs, which are very close to humans in urban and rural environments, being directly linked to the occurrence of visceral and cutaneous leishmaniasis in various municipalities in Sao Paulo State^3^. Dogs are also directly linked to urban leptospirosis, Chagas disease in rural environments, and toxoplasmosis^4-6^. Bovines are also a common source of infection and an important reservoir for diseases such as rural leptospirosis and Q fever, especially during the reproductive period, when the bacteria are eliminated by calving^2,6^. Cats are also important reservoirs, acting in the cycle of bartonellosis and toxoplasmosis^5,7^. Bats have adapted to both urban and rural environments, and their proximity to these reservoirs is common in Sao Paulo State. Vampire bats, for example, mainly feed on cattle herds, while insectivorous and frugivorous bats have a lot of contact with dogs and cats, since their main shelters are located in urban areas. The possibility of these bats acting as reservoirs and players in the cycle of these zoonoses is something to be investigated and debated among researchers^1^.

This review aims to explore the prevalence of zoonoses in Sao Paulo State, Brazil, focusing on the role of bats and the contribution of molecular biology and techniques such as polymerase chain reaction (PCR) to the diagnosis.

### Visceral leishmaniasis and cutaneous leishmaniasis

Leishmaniases are caused by the flagellated protozoan *Leishmania* spp. There are approximately 1.5 million cases of cutaneous leishmaniasis (CL) and 500,000 cases of visceral leishmaniasis (VL) per year. A population of 350 million is at risk, mainly distributed in tropical and developing countries. Ninety percent of global cases of VL occur in five countries, including Brazil. The same applies to CL, with seven countries, including Brazil, accounting for 90% of global cases. There are currently 20 species of *Leishmania* and 30 species of phlebotomine vectors. The main vectors that transmit the disease are female sandflies of *Lutzomyia*, popularly known as *mosquito-palha*. In Brazil, deforestation, encroachment into primary forests, mass rural-to-urban migration and the construction of dams have increased the incidence of the disease in recent years. These factors enabled a greater development of mosquito vectors, which is undoubtedly associated with the country’s economic inequality^3,8^.

The main causative species of VL in Brazil is *L. infantum* (syn. *chagasi*), while for CL, they are *L. amazonensis* and *L. braziliensis,* distributed nationwide, and *L. guyanensis*, concentrated in the Northern region. In humans, VL symptoms range from fever, weight loss and anemia to more serious complications, such as splenosis and hepatomegaly. In turn, CL manifests ulcerative lesions on the skin and mucous membranes, coryza, nosebleeds and sore throats^9,10^.

In Sao Paulo State, 8,553 cases were reported from 1999 to 2019. Among these cases, 3,046 (35.6%) were confirmed as autochthonous, with 266 resulting in death; an 8.7% overall mortality rate. Among the 645 municipalities, 107 (16.6%) reported autochthonous human transmission during the period. A total of 25.7% of the municipalities confirmed canine transmission and the presence of *Lutzomyia longipalpis* ^11^.

Bats are a natural food source for Phlebotominae, as they share the same habitats in caves and shelters, as well as being reservoirs for various species of Trypanosomatidae. In Brazil, there are environmental factors that favor the development of these vectors, as Brazil is a tropical country with high temperatures and excessive organic matter^12^.

A recent Brazilian study evaluated the detection of *Leishmania* species in bat species from their first detection to the present day. Among all bats evaluated, 11.6% (251/2,167) were infected. In total, 63 different bat species were analyzed, and *Leishmania* spp. were found mainly in *Molossus rufus* (77.3%, 34/44), *Desmodus rotundus* (22.7%, 15/66), *Artibeus planirostris* (18.7%, 12/64), *Carollia perspicillata* (16.9%, 12/71), *Artibeus lituratus* (16.7%, 20/120), *Molossus* (14.9%, 53/356) and *Glossophaga soricina* (8.6%, 31/361)^13^.

*L. amazonensis* has already been identified in several bat species, as have *L. infantum* and *L. braziliensis*. The findings of protozoans in bats from endemic areas suggest a direct relationship with the disease cycle in these places, as is the case for studies in Mato Grosso, with 28.9% positive cases (70/242); Belo Horizonte, with 1.67% (2/120); Montes Claros, with 4.4% (11/247); Espirito Santo, with 4.8% (5/105); Campo Grande, with 22.5% (18/80); and Sao Paulo, with 3.1% (21/683). The most commonly used molecular methods involve the detection of specific genes via conventional and nested PCR^14-18^. Interestingly, in non-endemic areas, positive results were also found, such as in Rio Grande do Sul with 1.2% (5/402) and in the Triangulo Mineiro and Alto da Paranaiba areas, with 8% (36/448)^19,20^.

### Chagas disease

Chagas disease (CD) results from infection caused by the protozoan *Trypanosoma cruzi*, being characteristic of the American continent. There are approximately 10 million people infected, 25 million at risk of contracting the infection and approximately 20,000 deaths from the disease per year^4^. *T. cruzi* vectors are hematophagous bedbugs, popularly known as kissing-bugs and technically called triatomines. Three important species are currently described: *Triatoma infestans*, *Rhodnius prolixus* and *Triatoma dimediata*, which are widely distributed throughout South America^21^. Wild species of triatomines have an even wider distribution, reaching North America, and are important for the wild cycle of the disease. These vectors are nocturnal and transmit *T. cruzi* via feces during hematophagy^21^.

In Brazil, an overall 4.2% prevalence of *T. cruzi* infection has been estimated in rural populations, with highest rates in Minas Gerais and Rio Grande do Sul (8.8%), Goias (7.4%), the Federal District (6.1%), Sergipe (6.0%) and Bahia (5.4%). From 2000 to 2013, 1,570 cases of acute or chronic CD were recorded by municipal health agencies, whereas only four cases were confirmed in Sao Paulo State during the entire period^22^.

*T. cruzi* vectors are widely present in homes and peridomiciles, as well as caves and shelters, and can have close contact with bats, either via feeding in the case of insectivorous bats or via contact with the feces of bedbugs. A Brazilian study in Acre State, in the Amazon region, analyzed 367 bats of different species and detected *Trypanosoma* in 81 samples. Among these strains, 16.5% (60/367) were *T. cruzi* ^23,24^. Acre, like Sao Paulo, has few recorded human cases of CD in the last decade. Another study in the same region detected 20.3% (15/74) positive cases^25^. A research group assessed the presence of *T. cruzi* in bats from different regions, including Para, Rio de Janeiro, Mato Grosso do Sul and Piaui, targeting species from all biomes, and the agent was detected in bats from all locations, resulting in 14% (13/93) positivity^26^. In Sao Paulo, a recent study detected 2.97% (6/20) positive cases in the hearts and kidneys of urban bats and vampire bats^27^. In addition to other parasitic diseases, CD is closely related to social, economic, political and cultural factors^28,29^.

### Toxoplasmosis

Toxoplasmosis is a parasitic disease caused by the protozoan *Toxoplasma gondii*. This protozoan is spread worldwide, infecting approximately one-third of the population^30^. Infection occurs mainly by consuming food or water contaminated by oocysts released in the feces of animal hosts. There has been much discussion about the complications that occur when pregnant women are infected during pregnancy and the risk factors associated with it^31^. Toxoplasmosis control is extremely complex and requires attention to all variables, including the role of wildlife in maintaining endemicity^30^.

The burden of toxoplasmosis in children born with congenital infection is very high^32^. Among congenitally infected children, several do not survive long after birth; 35% of them face neurological problems such as hydrocephalus, microcephaly and intellectual disabilities; 80% have eye damage; and 40% suffer hearing loss. The severity of clinical toxoplasmosis in Brazilian children may be related to the genetic characteristics of *T. gondii* strains prevalent in animals and humans in Brazil^5^.

The number of wild species infected by *T. gondii* is extensive. The routes of infection are associated with predation on intermediate hosts and consumption of contaminated vegetables or water^5^. In relation to bats, some suspicions have been raised, such as the consumption of fruit or water contaminated with oocysts or by vertical transmission, although few studies have proposed a real possibility according to the pathogenesis of the protozoan. The parasite has tissue tropism to the brain, heart and pectoral muscle of bats^33^. A study of bats in Sao Paulo city found that 32.6% (201/616) were seropositive for *T. gondii*, demonstrating exposure of local bats^34^. Studies have highlighted the potential role of bats in the toxoplasmosis cycle in Brazil. Research conducted in Natal city found that 21.6% (11/51) of bat tissue samples tested positive for *Toxoplasma gondii* using conventional PCR across different organs. Similarly, a study in Bahia reported 2.1% (2/97) positive results in bat brain samples using the same method^35,36^. *T. gondii* has already been genotyped in bat species from Sao Paulo city in non-hematophagous and hematophagous bats, pointing to the previous presence of these genotypes in other animal species, both domestic and wild, suggesting that this genotype is circulating in other Brazilian regions^34^.

### Bartonellosis

Bartonellosis is a bacterial disease caused by species of *Bartonella*, with different clinical forms, target hosts and pathogenic species. In Brazil, its most common zoonotic form, known as cat scratch disease (CSD), is caused by the species *B. henselae*. In cats, the infection prevalence is very high, reaching 90% in certain locations, making it difficult to differentiate infection from disease in some cases^37^. This high prevalence can be explained by persistent bacteremia in asymptomatic reservoir hosts, which is common and can facilitate the agent’s transmission process^38^. Bacterial transmission between cats occurs via fleas of the species *Ctenocephalides felis*, via inoculation, while transmission between cats and humans occurs via scratches or bites^37^. Ticks have also been studied as potential transmitters of *B. henselae* to humans^37^.

In cats, the disease is generally asymptomatic^39^. In humans, CSD is also asymptomatic, but when it persists in the body, it can develop into recurrent fever, endocarditis, encephalopathy in children, and osteomyelitis^37^.

In the United States, where the disease is frequently investigated, more than 40,000 cases of CSD and around 2,000 hospitalizations are reported per year^40^. In Brazil, there are few data available on the occurrence of cases at the population level. The distribution of samples related to bartonellosis in humans in Brazil from 2011 to 2017 indicates that 37.94% (480/1265) of samples were positive for anti-*B. henselae* antibodies. In terms of region, Southeast China was the most reactive, accounting for 85.42% (410/480) of the cases, with 31.04% (149/480) in Rio de Janeiro and 29.38% (141/480) in Minas Gerais. Compared with the rural population, the urban population was the most affected^41^.

A study of bat samples from five Brazilian states (Mato Grosso, Sao Paulo, Parana, Para and Tocantins), aimed at detecting the nuoG gene by real-time PCR, revealed *Bartonella* positivity in 5.3% (17/322) of liver, spleen and blood samples. None of the samples sequenced were *B. henselae*, but rather *B. taylorii* and *B. koehlerae* ^7^. Real-time PCR was also used to investigate the bacteria in bats from Maranhao, and of the 29 samples tested, none were positive^42^. Another study, in the states of Rio de Janeiro, Santa Catarina and Bahia, evaluated the presence of *Bartonella* in 18.5% (22/119) of the spleen samples via conventional PCR (gltA gene), but only at the genus level^43^. In another study with samples from Rio de Janeiro, 3.6% (4/110) of liver and spleen samples were positive by conventional PCR (gltA gene)^44^. When the presence of *Bartonella* in bat ectoparasites was assessed, 19.8% (40/202) of the flies were positive, whereas no mites out of 100 were positive according to qPCR (nuoG gene)^45^. A large study with samples from fifteen Brazilian states investigated the presence of the bacterium in the liver of hematophagous bats via real-time PCR combined with conventional gltA PCR. They reported 24.5% (51/208) positive samples, and after sequencing, the only species found were *B. bacilliformis* and *B. ancachensis* ^46^.

### Q fever

Q fever is a febrile human disease caused by the bacterium *Coxiella burnetii*. This pathogen can infect vertebrate and invertebrate animals and cause a disease called coxielosis, characterized by reproductive disorders^47^. This is a highly transmissible and infectious bacterium, and its main and most important route of transmission is airborne, in which the bacterium is dispersed in the environment after calving or animal abortion, contaminating it and making animals and humans close to the area susceptible^48^. From its aerosolization site, the bacterium can be carried by the wind for up to 30km, reaching populations that are not necessarily in direct contact with infected animals^48^.

In animals, reproductive complications such as abortions, stillbirths and premature births can occur^47^. In humans, most cases are asymptomatic; however, approximately 35 to 39% of patients can develop fever, body aches and headaches, and approximately 1 to 5% of patients usually develop more serious complications as a result of bacterial persistence in the body, such as pneumonia, hepatitis and endocarditis^47^. Although the percentage of persistent infections is minimal, the prevalence of the disease in Brazil does not seem to be, and cases of *C. burnetii* endocarditis have already been diagnosed in Brazilian hospitals, which suggests a much larger number of chronic diseases that have not yet been associated with the etiological agent^47^.

Sao Paulo is the largest Brazilian state with studies and reports of the disease, including an outbreak in slaughterhouse workers in the municipality of Barbosa (21°16’00”49°56’57”), and a seropositivity of 21.5% (129/604) in febrile patients suspected of having dengue in municipalities in the countryside^47^.

Bats are considered potential reservoirs of the disease since they are mammals with an aerial cycle and often have rural environments as natural habitats. The only study involving Q fever in bats in Brazil was carried out with samples from the states of Rio de Janeiro, Santa Catarina and Bahia. A total of 119 spleen samples were evaluated via conventional PCR (IS1111 gene), and 3.4% (4/119) were positive^43^. In Colombia, a group studied the presence of the bacterium in the blood of bats captured from caves and detected 6.3% (8/126) of positive bacteria by qPCR (IS1111 gene). The absence of human cases in the country also makes possible associations difficult^49^.

### Brazilian spotted fever

Brazilian spotted fever (BSF) is a serious febrile disease caused by the bacterium *Rickettsia rickettsii* and transmitted by *Amblyomma* ticks. This tick can be present on cattle, horses, dogs, poultry and some synanthropic animals; however, the main reservoirs of *Rickettsia* are capybaras^50^. In humans, the disease can cause fever, reddish patches on the body, muscle and joint pain, severe headache, nausea and neurological disorders. In animals, the disease is accompanied by fever, severe weight loss, hemorrhages, difficulty breathing and convulsions. Capybaras, in particular, are resistant to the disease, and thus are the preferred targets for ticks, being an epidemiological danger to humans^50^.

In Brazil, sporadic cases of BSF have been reported since it was first described, in 1929, until 1980. From 2000 to 2018, after a major effort by the Ministry of Health, 2090 cases were confirmed, 55% of which resulted in death. Sao Paulo ranked first among all Brazilian states, with 982 cases (47%). The Central-eastern region of Sao Paulo is considered an endemic area for BSF^51^.

Many tick infestations have already been described in bats, including the presence of mutual infection of zoonotic agents in bats and their parasites, as in the case of rickettsiae pathogenic to humans found in bats and their ticks in Sweden^52^. A study of bats in Sao Paulo city evaluated *R. rickettsii* seropositivity, showing that 8.6% (39/451) of them were seropositive^53^. In the states of Rio de Janeiro, Santa Catarina and Bahia, a study of 119 total spleen samples revealed no positives^43^. Also in Sao Paulo city, an interesting report of infection of ticks that feed on bats and humans has been published; however, it is a *Candidatus R. paranaensis* species^54^. The same occurred in Rio de Janeiro, with the sequencing of a positive sample for *Rickettsia* spp. in bat flies, in which similarity was found with *Candidatus R. andaenae* ^44^. Molecular evidence of *Rickettsia* spp. species in bat tissues has not yet been described in Brazil, but few studies on this topic exist. There is a need for more studies with samples from other locations, including endemic areas in the Sao Paulo countryside, to draw more conclusions about the importance of bats in the BSF cycle.

### Leptospirosis

Leptospirosis caused by the bacteria *Leptospira*. is widely distributed worldwide. The main species associated with clinical cases in Brazil is *L. interrogans* and its serovars. Leptospirosis is considered a disease of global importance, affecting overcrowded urban areas to a greater extent; however, rural populations that deal directly with farm animals are also affected. Mortality from this disease is significant, being associated with pathogenic strains and lack of early clinical diagnosis^55^.

Dogs are the most affected by the disease and have the highest mortality rate. Acute renal failure is the most serious consequence, and is usually the cause of death. In farm animals, especially cattle, leptospirosis is more common in nature, leading to abortions and low milk production. In humans, leptospirosis acts as an acute disease, leading not only to nonspecific signs, such as fever and body aches, but also to rapid kidney damage, causing acute renal failure, which is the most common cause of death. In severe cases, respiratory arrest can occur due to lung involvement, caused by intense breathing difficulties^6^.

Animal reservoirs are undoubtedly essential for the occurrence of leptospirosis. As is the case of urban rats, bats carry numerous infectious agents. The role of rats in the epidemiology of the disease has been well defined, but the role of bats in maintaining and/or transmitting *Leptospira* spp. to other animals or to humans has not been defined. Some studies have investigated the influence of wildlife in this chain, but few studies involving bats exist. One study assessed the presence of the bacterium via conventional PCR (LipL32 gene) in the kidneys of bats in Sao Paulo city, finding 1.7% (6/343) pathogenic leptospires^56^. In Rio Grande do Sul, one study obtained higher positivity, with 39% (36/92) of pathogenic leptospires^57^.

In Table 1, we organized the data resulting from the molecular investigations carried out in Brazil to date to detect the zoonotic agents discussed in this article in urban and rural bats.

**Table 1:** Molecular investigations carried out on Brazilian bats to detect zoonotic agents.

Type of bat	Molecular prevalence	Brazilian states	Article
***Leishmania***			
Urban bats	11.6% (251/2167)	MG, SP, RJ, ES, MS, MA	Ratzlaff *et al.* ^13^
Urban and rural bats	28.9% (70/242)	MT	Castro *et al*.^14^
Urban bats	1.67% (2/120)	MG	Araújo *et al*.^15^
Urban bats	4.4% (11/247)	MG	Araújo *et al*.^15^
Urban bats	4.8% (5/105)	ES	Riva *et al*.^16^
Urban bats	22.5% (18/80)	MT	Rezende *et al*.^17^
Urban and rural bats	3.1% (21/683)	SP	Savani *et al*.^18^
Urban bats	1.2% (5/402)	RS	Ratzlaff *et al*.^19^
Urban bats	8% (36/448)	MG	Gómez-Hernandéz *et al*.^20^
***Trypanosoma cruzi***			
Urban bats	16.5% (60/367)	AC	Santos *et al.* ^24^
Urban bats	20.3% (15/74)	AM	Coura *et al*.^25^
Urban and rural bats	14% (13/93)	PA, RJ, MS, PI	Lisboa *et al*.^26^
Urban and rural bats	2.97% (6/20)	SP	França *et al*.^27^
***Toxoplasma gondii***			
Urban and rural bats	32.6% (201/616)	SP	Cabral *et al*.^34^
Urban bats	21.6% (11/51)	RN	Fournier *et al*.^35^
Urban bats	2.1% (2/97)	BA	Jesus *et al*.^36^
***Bartonella***			
Urban bats	5.3% (17/322)	MT, SP, PR, PA, TO	Ikeda *et al*.^41^
Urban bats	0% (0/29)	MA	Braga *et al*.^42^
Urban bats	18.5% (22/119)	RJ, SC, BA	Ferreira *et al*.^43^
Urban bats	3.6% (4/110)	RJ	Gonçalves-Oliveira *et al*.^44^
Bat ectoparasites	19.8% (40/202)	RJ	Amaral *et al*.^45^
Rural bats	24.5% (51/208)	All biomes	André *et al*.^46^
***Coxiella burnetii***			
Urban bats	3.4% (4/119)	RJ, SC, BA	Ferreira *et al*.^43^
***Rickettsia rickettsii***			
Urban bats	8.6% (39/451)	SP	Tompa *et al*.^52^
Urban bats	0% (0/119)	RJ, SC, BA	Ferreira *et al*.^43^
***Leptospira***			
Urban bats	1.7% (6/343)	SP	Bessa *et al*.^56^
Urban bats	39% (36/92)	RS	Mayer *et al.* ^57^

### Application of molecular biology in the diagnosis and determination of zoonoses in populations

Molecular biology is crucial for the diagnosis, monitoring and prevention of zoonoses in populations. The ability to rapidly identify pathogens, trace their genetic evolution and understand transmission dynamics is essential to protect public health and minimize zoonotic impact^58^.

Compared with conventional diagnostic tools, molecular tools enable the etiological agent within a population to be diagnosed with greater efficiency and precision. These tools have increased the understanding of the relationship with the host, pathogenicity and virulence of etiological agents, provided information on their genetic structure and taxonomy, and enabled the determination of the zoonotic potential of previously unidentified agents. Molecular tools have also provided a dimension to disease epidemiology that would be unattainable via conventional diagnostic tools. Molecular epidemiology was defined as a science that focuses on the contribution of potential genetic and environmental risk factors, identified at the molecular level, to the etiology, distribution and prevention of diseases within families and between populations^59^.

Molecular biology enables sensitive and specific detection of pathogens. This is accomplished via techniques such as PCR, which amplifies the genetic material of a pathogen, making it easier to identify. The development of nucleic acid amplification techniques led to further improvements in the identification and taxonomy of microorganisms. The main advantages of these techniques are speed, increased discriminatory power and the ability to analyze small amounts of sample^59^. An important advantage of PCR-based procedures is their ability to detect and characterize the genetic variability of infectious agents, particularly at the intraspecific level. In addition, PCR tests, if properly designed, can enable the direct detection of parasite DNA from mixed templates, such as clinical or environmental samples, quickly and with high levels of sensitivity and specificity^58^.

Some specific methods are commonly applied in the diagnosis of the zoonoses discussed in this review, and have shown good results in direct diagnosis in bat tissues. Table 2 shows the main methods, gene targets and oligonucleotides used for molecular diagnosis in bat tissues.

**Table 2:** Main methods, gene targets and oligonucleotides used for the molecular diagnosis of zoonotic agents prevalent in Sao Paulo State in bat tissues.

Zoonotic agent	Method	Target gene	Oligonucleotide	Article
*Leishmania*	cPCR	IRBP	IRBPfw (TCC AAC ACC ACT GAG ATC TGG AC); IRBPrev (GTG AGG AAG AAA TCG GAC TGG CC)	Castro *et al*.^14^
	cPCR	ITS-1	LITSR (CTG GAT CAT TTT CCG ATG); L58S (TGA TAC CAC TTA TCG CAC TT).	Araújo *et al*.^15^
	cPCR	kDNA	D1 (GGG GAG GGG CGT TCT GCG AA); D2 (CCG CCC CTA TTT TAC ACC AAC CCC)	Riva *et al.* ^16^
	nPCR	SSU rDNA	S4 (GAT CCA GCT GCA GGT TCA CC); S12 (GGT TGA TTC CGT CAA CGG AC); S17 (CCA AGC TGC CCA GTA GAA T); S18 (TCG GGC GGA TAA AAC CC)	Ratzlaff *et al*.^17^
*Trypanosoma cruzi*	cPCR	mini-exon	TCZ1 (CGA GCT CTT GCC CAC ACG GGT GCT); TCZ2 (CCT CCA AGC GGA TAG TTC AGG)	Lisboa *et al*.^26^
	cPCR	kDNA	121 (AAA TAA TGT ACG GGT GAG ATG CAT GA); 122 (GGG TTC GAT TGG GGT TGG TGT)	França *et al*.^27^
*Toxoplasma gondii*	cPCR	B1	T1 (AGC GTC TCT CTT CAA GCA GCG TA); T2 (TCC GCA GCG ACT TCT ATC TCT GT)	Fournier *et al*.^35^
	cPCR	B1	TOX4 (CGC TGC AGG GAG GAA GAC GAA AGT TG); TOX5 (CGC TGC AGA CAC AGT GCA TCT GGA TT)	Jesus *et al*.^36^
*Bartonella*	qPCR	nuoG	F-Bart (CAA TCT TTT GCT TCA CC); R-Bart (TCA GGG CTT TAT GTG AAT AC)	Ikeda *et al*.^41^
	cPCR	gltA	F (GCT ATG TCG CVT TCT ATC AYG A); R (AGA ACA GTA AAC ATT TCN GTH GG)	Ferreira *et al*.^43^
*Coxiella burnetii*	qPCR	IS1111	F (TAT GTA TCC ACC GTA GCC AGC); R (CCC AAC ACC TCC TTA TTC)	Ferreira *et al*.^43^
*Rickettsia rickettsii*	cPCR	gltA	F (AAA TGA GTA TTT TTA GTA GG); R (AAG GTT TAT CCG TTT AGG CT)	Tompa *et al*.^52^
*Leptospira*	qPCR	Lipl32	45F (AAG CAT TAC CGC TTG TGG TG); 286R (GAA CTC CCA TTT CAG CGA TT)	Mayer *et al*.^57^

In addition to PCR, nucleic acid sequencing makes it possible not only to confirm the presence of the agent, but also to identify mutations and genetic variations in pathogens that can affect transmission and virulence. It rapidly provides large amounts of genetic information and tends to become one of the most important pillars in the routine of public health laboratories^60^. Sequencing was initially expensive and time-consuming, especially when it was performed manually, but with the development of cycle sequencing and automated systems, cost and time have greatly decreased, making large-scale sequencing faster and more accessible^60^. Direct sequencing of a PCR product is commonly used to detect pathogens; however, if there are multiple related sequences in an amplification product, either due to mixed infection with similar genotypes or due to polymorphic alleles, deciphering the sequence may be impossible, or the mixed template may be completely lost by preferential amplification. Cloning the template can overcome this problem, but this is a laborious process, and variant molecules can still be lost if they exist at low frequencies^58^.

Small amounts of information from sequencing can be used to identify pathogens at the species level, even if primers are used at the genus or family level. On the other hand, large amounts of information are essential to perform a phylogenetic analysis^58^. This analysis is crucial for the study of molecular epidemiology, as it provides a solid understanding of the taxonomy and population structure of the microorganism, which is essential to study aspects of the pathogen’s biology and epidemiology, including its host specificity, genetic diversity and zoonotic potential^60^. PCR sequencing in combination with phylogenetic analysis has been used to investigate the biology of a variety of agents, as well as to identify the relationship of agents detected in different geographical areas and the genetic proximity between them^60^.

Another advance in the application of molecular techniques to epidemiology has been the development of technology to measure nucleic acid amplification in real time, such as qPCR, to quantify the number of parasites in a given sample or measure gene expression levels^58^. A variety of chemicals can be used to detect nucleic acid amplification in real time, including SYBR Green dyes and Taqman probes. Real-time amplification has several advantages over conventional amplification, including simple DNA quantification, high-speed analysis and high-throughput analysis. Real-time PCR is much more sensitive than conventional PCR, detecting fragments that cannot be detected by conventional equipment^58^. The ability of qPCR to perform analysis in closed tubes eliminates the need for post-PCR handling, such as agarose gel electrophoresis, reducing the risk of contamination and reducing sample handling, while also reducing costs^59^. In the case of probe-based systems, it is possible to detect single nucleotide polymorphisms, which greatly increases sensitivity and confirms the agent at species level without the need for genetic sequencing. With real-time molecular approaches, it is possible to obtain more information in genotyping, as it can theoretically detect and differentiate all mutants at different base positions within the probe’s binding region, which enables tracing the origin and spread of zoonoses in populations^59^.

PCR-based techniques may be the only means of diagnosis to uncover the role of animals as mechanical or zoonotic transmitters of diseases^60^. It is imperative that, in situations such as these, simultaneous surveys are carried out in human and animal populations to correlate data at the epidemiological and molecular levels and to rule out the possibility of geographical variation. A good choice of genetic markers according to the epidemiological issue being investigated is essential for greater sensitivity in detecting the agent present in the study area, making the technique better suited to the epidemiological context. A robust and well-designed PCR is an ideal diagnostic tool for epidemiological studies aimed at uncovering zoonotic relationships^58^.

A better accuracy of diagnostic tests invariably results in a greater degree of confidence for epidemiological statistics and provides another dimension to the study of microbial epidemiology^58-60^.

## CONCLUSION

This study summarizes the importance of understanding the complex dynamics of the zoonoses discussed and the possible role of bats in maintaining these diseases in Brazil. The prevalence and epidemiology of these diseases highlight the urgent need for effective control and prevention measures, especially in Sao Paulo State, where interactions between humans and bats are significant. Studies focusing on bats as natural reservoirs reveal the relevance of these animals in the chain of transmission of various pathogens. In addition, the application of molecular biology techniques in the diagnosis of these zoonoses is essential for early and accurate detection, enabling rapid and targeted interventions. The integration of ecological and molecular approaches is, therefore, fundamental to mitigate these diseases and promote public health.
